# Molecular Dynamics Simulation on B3-GaN Thin Films under Nanoindentation

**DOI:** 10.3390/nano8100856

**Published:** 2018-10-19

**Authors:** Chen Chen, Haitao Li, Henggao Xiang, Xianghe Peng

**Affiliations:** 1 Department of Engineering Mechanics, College of Aerospace Engineering, Chongqing University, Chongqing 400044, China; chchen_cqu@aliyun.com (C.C.); xhpeng@cqu.edu.cn (X.P.); 2 Chongqing Key Laboratory of Heterogeneous Material Mechanics, Chongqing University, Chongqing 400044, China

**Keywords:** molecular dynamics, B3-GaN, anisotropy, prismatic loops, dislocation density

## Abstract

The B3-GaN thin film was investigated by performing large-scale molecular dynamics (MD) simulation of nanoindentation. Its plastic behavior and the corresponding mechanism were studied. Based on the analysis on indentation curve, dislocation density, and orientation dependence, it was found that the indentation depths of inceptive plasticity on (001), (110), and (111) planes were consistent with the Schmid law. The microstructure evolutions during the nanoindentation under different conditions were focused, and two formation mechanisms of prismatic loop were proposed. The “lasso”-like mechanism was similar to that in the previous research, where a shear loop can translate into a prismatic loop by cross-slip; and the extended “lasso”-like mechanism was not found to be reported. Our simulation showed that the two screw components of a shear loop will glide on another loop until they encounter each other and eventually produce a prismatic dislocation loop.

## 1. Introduction 

Group-III metal nitrides are a kind of interstitial compounds, which have caused extensive concern in the past decade. For example, high-temperature metal nitride coatings have many applications in cutting tools, wear-resistant parts, and jewelry industries [1]. Among which, gallium nitride (GaN) is a wide-band gap semiconductor and is supposed to be one of the most popular candidates because of its dystectic point, high frequency, and high-power [2]. On account of these outstanding properties, GaN has been widely used in piezoelectric and thermoelectric materials [3]. It is well known that there are four kinds of crystal structures in GaN, including the NaCl-type structure (B1) under high pressure, the zinc-blende structure (B3) under unbalanced deposition parameters, the honeycomb-type structure [4], and the wurtzite structure (B4) under atmospheric pressure. In some respects, the properties of B3-GaN are better than that of B4 structure. For example, B3-GaN can achieve higher saturation and electron drift speed, and smaller band gap than B4 structure [5]. With the advance of preparation technology, the B3-GaN can be fabricated with the modified surface-activated-bonding (SAB) method at room temperature [6]. When this promising semiconductor material is in service, it may be subjected to pressure, inducing micro-structural deformation. Therefore, it is necessary to have a detailed understanding of the deformation behavior. 

Until now, much effort has been made to investigate the properties of GaN films by experiments and first-principle calculations. For example, Mishra et al. found nanoflowers on the surface of GaN films and analyzed surface chemistry and electronic structure [7]. Herval et al. studied the interface imperfections of cubic GaN multiple films using temperature-dependent photoluminescence and found three different configurations at interfaces [8]. Gao et al. investigated the crystalline structural formation in GaN at three different cooling rates [9]. However, the research on the structural deformation in GaN films is not enough, particularly for the B3 structure. Their mechanical properties need to be further investigated.

To characterize the mechanical behavior of nano-structures, the methodologies of continuum mechanics can be exploited to study the size-effects. For example, an innovative stress-driven nonlocal integral elasticity model was proposed for the size-dependent analysis, which can avoid the difficulties of the classical strain-driven nonlocal law [10]. Nevertheless, these methods cannot solve some issues, such as how a prismatic loop forms and propagates. Thus, molecular dynamics (MD) simulation is one good choice. Employing MD simulation, we carried out nanoindentation on B3-GaN (001), (110), and (111) planes to study the structural evolutions, orientation dependence, and prismatic loop formation. The corresponding mechanical response and dislocation density were also analyzed. In Section 2, the details of simulation, potential function, and methods to identify nanostructure are illustrated. In Section 3, the results of the simulation are presented and discussed, and in Section 4, the conclusion is drawn and presented.

## 2. Method

### 2.1. Selection of Potentials

The potentials are used to simulate the interatomic interaction. In our work, we selected the Stillinger-Weber (SW) potential [11] by Serra et al. [12] to describe the Ga-N system. The SW potential takes into account two-body and three-body interactions, and can well describe the total energy of a system, which is expressed as described in [13]

(1) $$\Phi \left(1,\ldots ,N\right)=\sum_{\begin{array}{l} i,j\\ \left(i<j\right) \end{array}}\varphi_{2}\left(i,j\right)+\sum_{\begin{array}{l} i,j,k\\ \left(i<j<k\right) \end{array}}\varphi_{3}\left(i,j,k\right)$$

where

(2) $$\varphi_{2}\left(r_{ij}\right)=\varepsilon f_{2}\left(r_{ij}/l\right),\;\varphi_{3}\left(r_{ij}\right)=\varepsilon f_{3}\left(r_{ij}/l\right)$$

(3) $$f_{2}\left(r\right)=\{\begin{array}{c} A\left(Br^{-p}-r^{-q}\right)\exp\left[\frac{1}{r-d}\right]\\ 0,\begin{array}{cc} & r>d \end{array} \end{array},\begin{array}{cc} & r<d \end{array}$$

(4) $$f_{3}\left(i,j,k\right)=h\left(r_{ij},r_{jk},\theta_{ijk}\right)+h\left(r_{ji},r_{ik},\theta_{jik}\right)+h\left(r_{jk},r_{ki},\theta_{jki}\right)$$

(5) $$h\left(r_{ij},r_{jk},\theta_{ijk}\right)=\exp\left[\gamma {\left(r_{ij}-d\right)}^{-1}+\gamma {\left(r_{jk}-d\right)}^{-1}\right]{\left(\cos\theta_{ijk}+\frac{1}{3}\right)}^{2}$$

where *A*, *B* and *γ* are bond strength parameters, *ε* and *l* are the energy and length units, *d* is the cut-off distance, *θ_ijk_* is the angle between the *ij* and *jk* bonds. *p* = 4 and *q* = 0.

To confirm the reliability of this potential for B3-GaN, the basic parameters, such as bulk modulus, lattice constant, cohesive energy, and elastic constants, are calculated with MD simulation and compared to those obtained by experiments or density functional theory (DFT). The results are listed in Table 1. It should be noted that the reliability of potentials can also be tested by comparison with material properties obtained at finite temperature via ab initio MD simulations [14,15,16]. In general, one should not expect that the predictions of classical MD [17] match perfectly well with those of ab initio MD [18].

The fundamental properties of B3-GaN obtained by MD are well fitted to experiments or DFT, so SW potential can be used to describe the interatomic interaction for the Ga-N system.

### 2.2. Simulation Details

**Figure 1:**
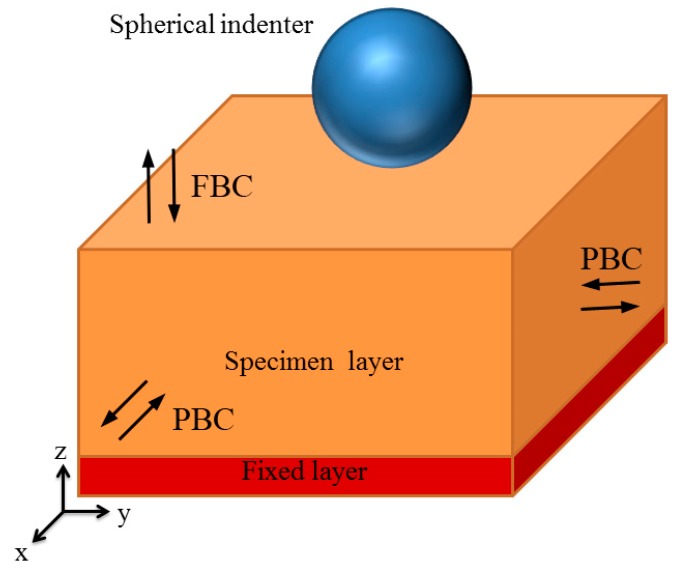
Set-up for indentation on GaN film with spherical indenter.

There are three different indentation planes to be investigated on GaN films: (001), (110), and (111) planes. The size of the simulation box was about 253 Å × 253 Å × 303 Å, comprising approximately 1,128,960 atoms. To optimize the specimen, we used the conjugate gradient (CG) algorithm to obtain the minimum equilibrium energy. The bottom atoms of the specimen were fixed to avoid movement during indentation, as shown in Figure 1. To perform the indentation simulation at a prescribed temperature, the Langevin thermostat was employed. In our simulation, the temperature was kept at 10 K for most cases. The indenter was set as a rigid sphere to save computation time, and it repels all atoms it contacts. The interaction potential and corresponding parameters can be found in the literature [25]. Before indentation, the indenter was placed 2 Å above the specimen. It moved downwards at a speed of 20 m/s. The periodic boundary conditions (PBC) were applied in the directions of the *X* and *Y* axes, and the fixed boundary condition (PBC) was applied in the *Z*-direction. The MD simulation was carried out using Large-scale Atomic/Molecular Massively Parallel Simulator (LAMMPS) (Sandia National Laboratory, Albuquerque, NM, USA) [26], with a constant time step of 1 fs, and the simulation results were visualized with OVITO (Version 2.9.0, Alexander Stukowski, Darmstadt, Germany) [27].

### 2.3. Structure Identification 

To analyze the structural deformation and dislocation nucleation, the identify diamond structure (IDS) method [28] was employed, which can be used to distinguish crystal structure [29]. In addition, to identify dislocation patterns, the dislocation extraction algorithm (DXA) [30] was adopted. DXA can effectively identify perfect dislocation and partial dislocation and calculate the length of dislocation lines.

## 3. Results and Discussions

### 3.1. Deformational Behavior of GaN under Indentation

**Figure 2:**
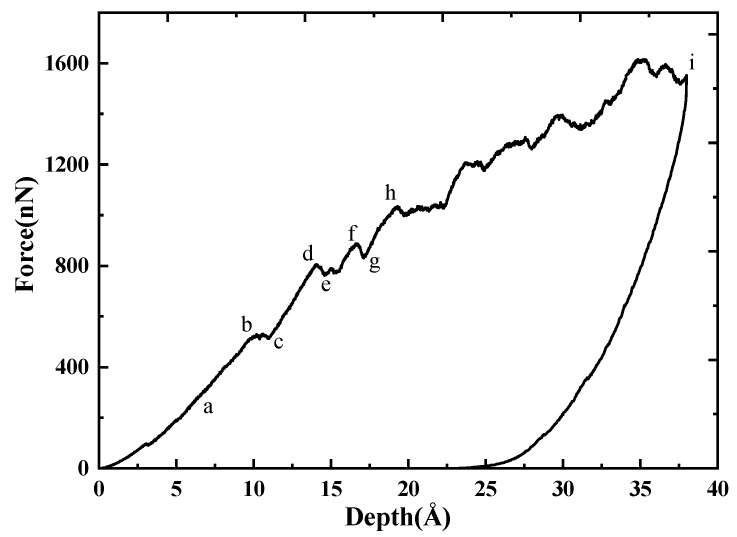
*P*-*h* curve of indentation on GaN (001) plane.

To further understand the deformation mechanism, the indentation on GaN (001) plane was performed. Figure 2 shows the indentation force-depth (*P*-*h*) curve. The loading curve and unloading curve are different, which indicates that there was plastic deformation during the loading process. To study the deformation clearly, we chose some special points on the curve, which are Points a (*h* = 7.0 Å), b (*h* = 12.2 Å), c (*h* = 13.0 Å), d (*h* = 14.0 Å), e (*h* = 14.7 Å), f (*h* = 16.7 Å), g (*h* = 17.1 Å), h (*h* = 19.3 Å) and i (*h* = 38.0 Å). During the loading, the curve can be divided into four stages by Points b, d, and h, which are (I) *h* < 12.2 Å; (II) 12.2 Å < *h* < 14.0 Å; (III) 14.0 Å < *h* < 19.3 Å; (IV) 19.3 Å < *h* < 38.0 Å.

**Figure 3:**
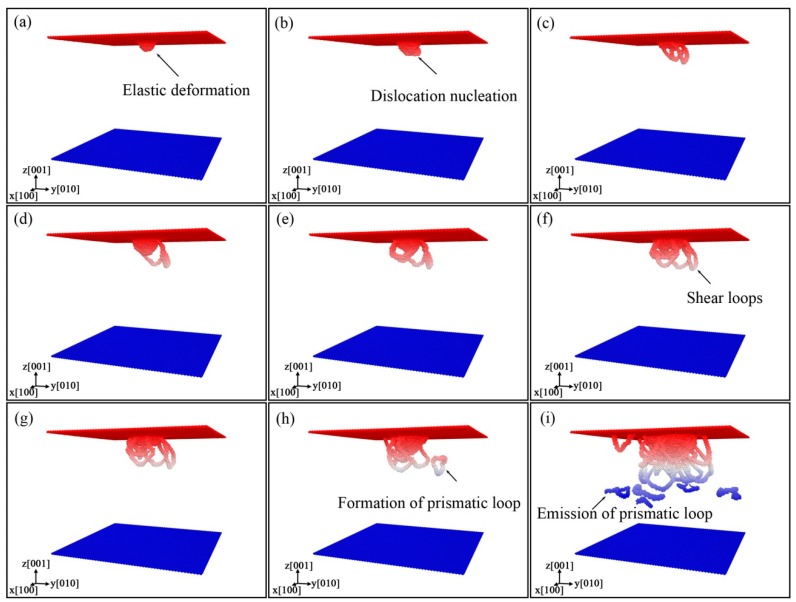
Distribution of dislocations in the specimen at different *h*: (**a**) *h* = 7.0 Å, (**b**) *h* = 12.2 Å, (**c**) *h* = 13.0 Å, (**d**) *h* = 14.0 Å, (**e**) *h* = 14.7 Å, (**f**) *h* = 16.7 Å, (**g**) *h* = 17.1 Å, (**h**) *h* = 22.3 Å, (**i**) *h* = 38.0 Å, with atoms colored by depth.

At Point a in Stage (I), it can be observed that the deformation occurs in the contact region between indenter and specimen, and the deformation is elastic, as shown in Figure 3a. Between Points b and c, there is a platform on the curve, corresponding to dislocation nucleation (Figure 3b), which may induce the redistribution of the local configuration and partly release the energy stored. A sudden pop-in occurs between Points d and e, which implies that a new dislocation nucleates, as shown in Figure 3e. With the increase of *h*, more dislocations nucleate and grow, inducing the drop of stress (e.g., between the Points f and g) and the release of strain energy. The *P*-*h* curve in Stage (IV) increases in a zigzag way. In this stage, the dislocations nucleate and interact with each other, and many dislocations appear. With the rise of dislocations in different directions, the dislocation density increases, and the prismatic loops are formed and emitted, as shown in Figure 3g–i. Using the DXA method, one can find that there are only perfect dislocations with the Burgers vector **b** = 1/2<110>.

During the indentation, the distribution of stress will affect the deformation of the nanostructure. The atomic stress can be expressed as described in [31],

(6) $$\sigma_{mn}=\frac{1}{N_{R}}\sum_{i}\left[\frac{m_{i}v_{i}^{m}v_{i}^{n}}{V_{i}}-\frac{1}{2V_{i}}\sum_{j}\frac{\partial \varphi \left(r_{ij}\right)}{\partial r_{ij}}\frac{r_{ij}^{m}r_{ij}^{n}}{r_{ij}}\right]$$

where *N_R_* is the number of atoms in cutoff distance, *m_i_* is the mass, $r_{ij}^{}=\left|r_{ij}\right|$ is the distance between the atoms *i* and *j*, $r_{ij}^{m}$ and $r_{ij}^{n}$ are two components of vector $r_{ij}$. *V_i_* is Voronoi volume surrounding the atom *i* and is expressed as outlined in [32],

(7) $$V_{i}=\frac{4\pi }{3}{\left(\frac{\sum_{j}r_{ij}^{-1}}{2\sum_{j}r_{ij}^{-2}}\right)}^{3}$$

It is well known that dislocation can lead to plastic deformation, and plastic deformation is closely related to the Mises stress expressed as in [33],

(8) $$\sigma_{s}=\sqrt{\sigma_{11}^{2}+\sigma_{22}^{2}+\sigma_{33}^{2}-\left(\sigma_{11}\sigma_{22}+\sigma_{22}\sigma_{33}+\sigma_{33}\sigma_{11}\right)+3\left(\sigma_{12}^{2}+\sigma_{23}^{2}+\sigma_{31}^{2}\right)}$$

**Figure 4:**
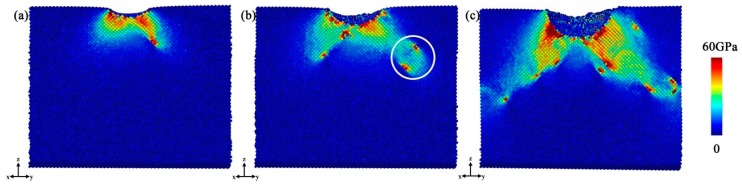
Distribution of Mises stress at different *h*: (**a**) *h =* 12.2 Å; (**b**) *h* = 22.3 Å; (**c**) *h* = 38 Å.

Figure 4 shows the distribution of Mises stress at different *h*. It can be found that the Mises stress field extends along the direction of <$0\bar{1}1$>, and dislocation nucleation that can be observed in Figure 3b also occurs synchronously at this depth. In Figure 4b, the Mises stress field sequentially extends along the direction of <$0\bar{1}1$>, and the local stress increase indicated by a white circle implies a detached prismatic loop observed in Figure 3h. When the indenter reaches the deepest position, the Mises stress field tends to be uniformly distributed along the 45° direction, which corresponds to Figure 3i. The distribution of stress is consistent with the distribution of the defect structure. When *P* is applied to the specimen, dislocation moves on the easiest slip plane, accompanied by the defect structures, so the Mises stress field extends along the slip direction.

**Figure 5:**
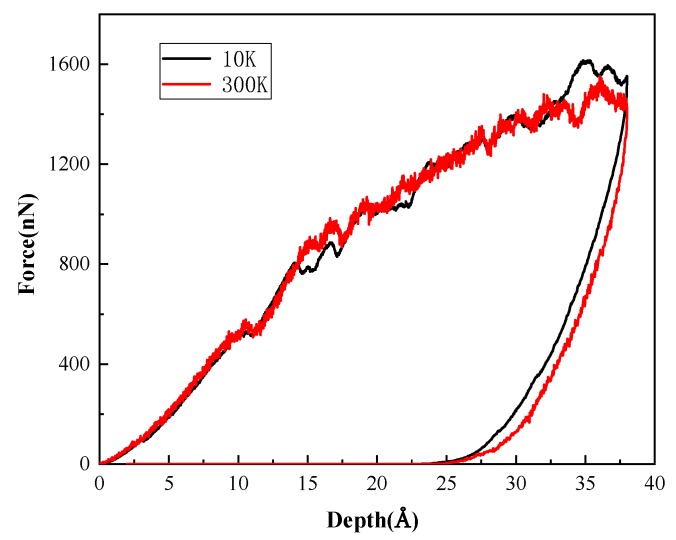
*P*-*h* curves at different temperatures.

The *P*-*h* curves of indentation on (001) plane at 10 K and at 300 K are shown in Figure 5. It can be found that the two curves have similar trends, but the curve at 10 K is smoother than that at 300 K, which is due to the more intense thermal motion of atoms at 300 K. It can also be found that the peak of curve at 300 K is slightly lower than that at 10 K, which indicates temperature influences the strength of the material.

### 3.2. Anisotropy in B3-GaN

**Figure 6:**
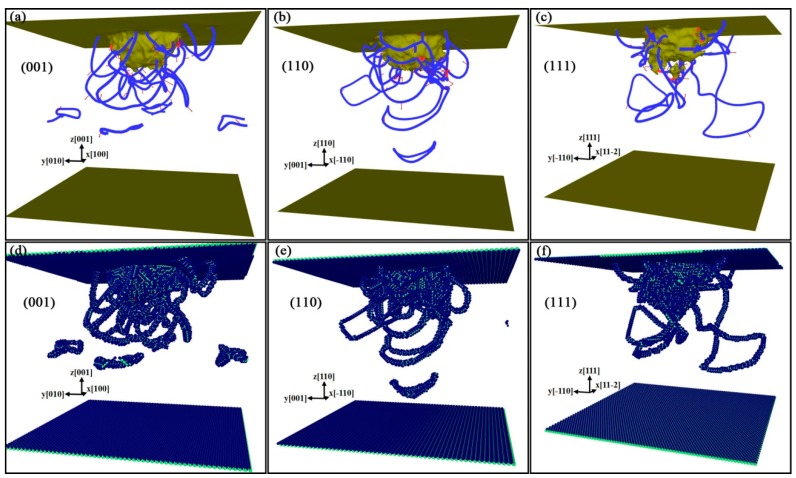
Dislocation distributions in GaN films indented on different planes at *h* = 35 Å. (**a**–**c**) DXA schematics in GaN films indented on (001), (110), and (111) planes, respectively, with dislocation lines indicated in blue, Burgers vectors by red arrows. (**d**–**f**) the corresponding IDS schematics.

To analyze the orientation dependence for the properties of the B3-GaN crystal, the indentations on (001), (110), and (111) planes were performed respectively. Figure 6 displays the dislocation distribution on different planes, obtained with IDS and DXA, respectively, at *h* = 35 Å. The indentations on the three planes have similar plastic responses, which are dominated by dislocation nucleation and emission of dislocation loop. There are many shear loops adhering to the indentation pit and prismatic loops emitted into the substrate. These loops are of perfect dislocations with Burgers vector **b** = 1/2<110>, as shown with the blue lines in Figure 6. This is reasonable, because in the B3 structure, the easiest slip direction is <110> [34]. However, the dislocation structures exhibit anisotropic for indentation on different surfaces. 

**Figure 7:**
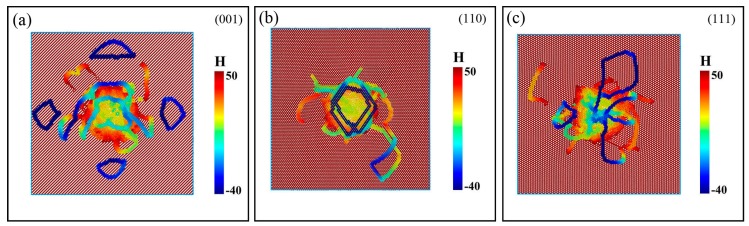
Upward view of films under indentation on different surfaces. (**a**), (**b**) and (**c**) represent the (001), (110) and (111) surfaces, respectively. H-value represents position of particles along *Z* direction.

As shown in Figure 6 and Figure 7, dislocations slip along <110> directions on {111} planes. This is in accordance with Peierls-Nabarro law, which can be expressed as in [35],

(9) $$\tau_{PN}=\frac{2G}{1-\nu }e^{-2\pi W/b}$$

where $W=\frac{c}{1-\nu }$ is the dislocation width, *G* and *v* are shear modulus and Poisson’s ratio, *c* represents the interplanar spacing, and *b* the interatomic spacing. Thus, the Peierls stress, an obstacle to the motion of dislocation, will decrease with the increase of *c* or with the decrease of *b*. For the B3 structure, the maximum interplanar spacing is among {111} planes and the close-packed directions are <110>, which is in accordance with the simulation results in the above figures.

**Figure 8:**
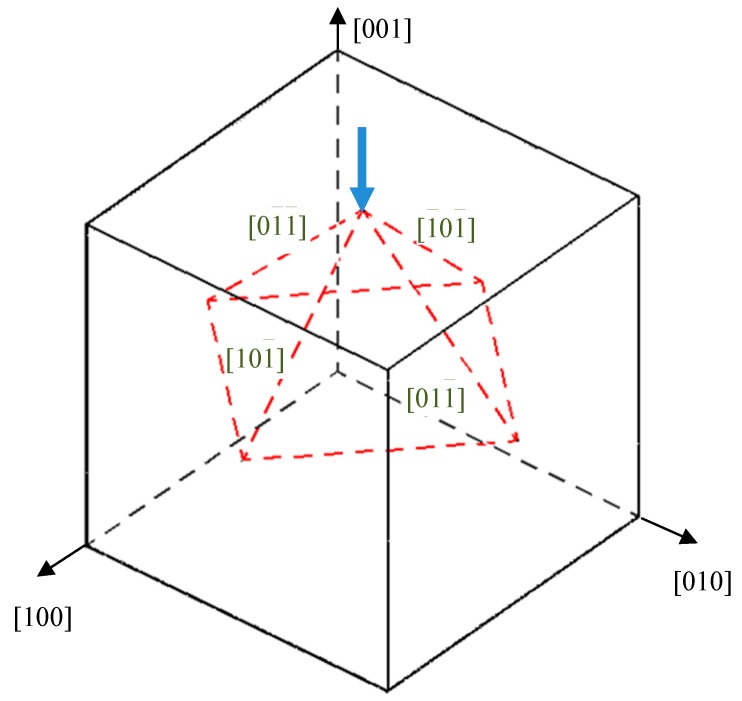
Sketch for slip systems under force along $\left[00\bar{1}\right]$.

It also can be seen that there are the largest number of prismatic dislocation loops in the case of indentation on (001) plane. These loops are emitted along the four oblique <110> directions and propagate in the substrate. It can be illustrated with the diagram in Figure 8. When the crystal is subjected to a force along $\left[00\bar{1}\right]$, dislocations will glide on {111} planes. Four slip directions, namely $\left[0\bar{1}\bar{1}\right]$, $\left[\bar{1}0\bar{1}\right]$, $\left[01\bar{1}\right]$ and $\left[10\bar{1}\right]$, can be activated simultaneously, thus we can see four prismatic loops with four-fold symmetry from top view or upward view, as shown in Figure 7. The indentation on (110) plane shows that the dislocation loops emitted move downward, perpendicular to the surface along **b** = 1/2<110> direction. For (111) plane, only some dislocation loops that attach to the indentation pit propagate along the direction of <110>.

**Figure 9:**
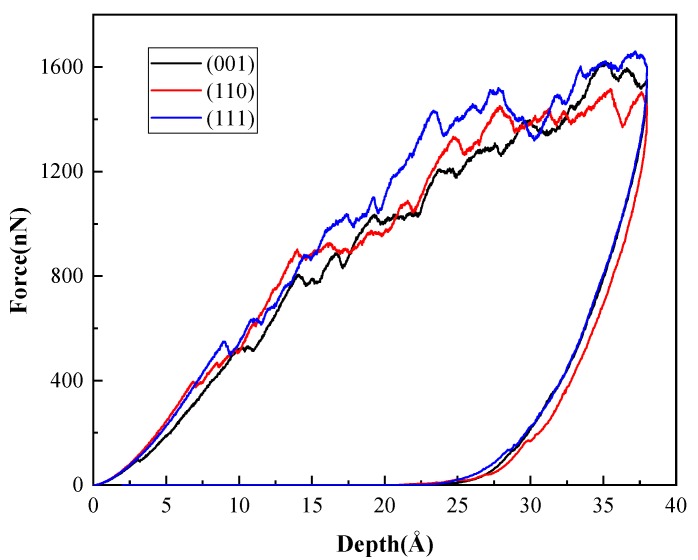
*P*-*h* curves of indentation on three B3-GaN surfaces. Inset shows distribution of dislocations in the sample under indentation on (111) plane as pop-in event happens.

Figure 9 displays the *P*-*h* curves for indentation on three different crystal planes. During loading, the three curves have a similar trend, accompanied with multiple pop-ins. It can be found that the pop-in is associated with the dislocation slip, as shown in the inset for the indentation on (111) plane, which is in accordance with the results observed in experiments [36,37,38,39] and simulations [34,25] of other materials. Comparing the three curves, we can also find that pop-in occurs first for indentation on (001) plane, followed by (110) plane and (111) plane. 

This phenomenon can be comprehended through the Schmid law. When an external force is applied on crystal, the resolved shear stress (RSS) on slip plane along slip direction is as described in [40],

(10) $$\tau_{RSS}=\sigma \cos\lambda \cos\phi =\sigma \mu$$

where *σ* is the applied stress, *μ* is Schmid factor, *λ* and *φ* are the angles between the loading directions and the slip plane normal and slip direction, respectively. If RSS reaches a critical value, namely CRSS, a slip is activated. Given CRSS is constant, it can be concluded that a larger Schmid factor will make dislocation slip easier. For (001) indentation shown in Figure 8, the angle *λ* is 54.7°and *φ* is 45°, so the Schmid factor is 0.408. Under (110) and (111) indentation, the Schmid factors are 0.405 and 0.273, respectively. It agrees with the degree of force in Figure 9, when the first pop-in occurs for indentation on different planes. It should be noted that during the indentation, the force exerted on the substrate atoms in the contact area is not always along the same direction due to the pit shape, and this may affect the value of the Schmid factor to some extent.

The hardness can also be obtained in the indentation simulation. Hardness is associated with the load and contact area, and is defined as in [41]:

(11) $$H=\frac{P_{\max}}{A_{c}}$$

where *P*_max_ represents the maximum indentation load, *A_c_* is the projected contact area calculated with the following equation,

(12) $$A_{c}=\pi \left(2R-h\right)h$$

where *h* is the indentation depth and *R* the radius of the indenter.

The calculated values of the hardness on (001), (110), and (111) planes are 30.9 GPa, 28.9 GPa and 31.9 GPa, respectively, so the (111) plane has the maximum hardness, which can be explained with that (111) plane is the close-packed plane, where slip is not easy to occur as the loading direction is perpendicular to it.

### 3.3. Formation and Propagation of Prismatic Loops

The frequently reported mechanism for the formation and propagation of prismatic loops is called “lasso”-like mechanism [42]. It indicates that the two screw components of the shear loop undergo cross-slip and interaction, and eventually form a prismatic loop. In our MD simulations, we find two kinds of mechanisms. One is “lasso”-like mechanism, and the other is more complex and can be called extended “lasso”-like mechanism.

**Figure 10:**
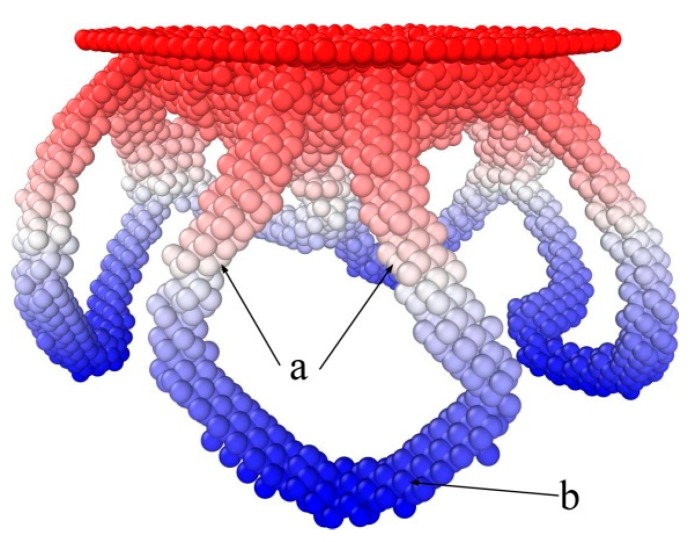
Dislocation loops during indentation, where “a” and “b” indicate screw and edge components, respectively.

The shear loops are made up of edge and screw segments, as shown in Figure 10. The screw segments in a loop are perpendicular to the indentation plane and have opposite signs; the edge segments are parallel with the indentation plane.

**Figure 11:**
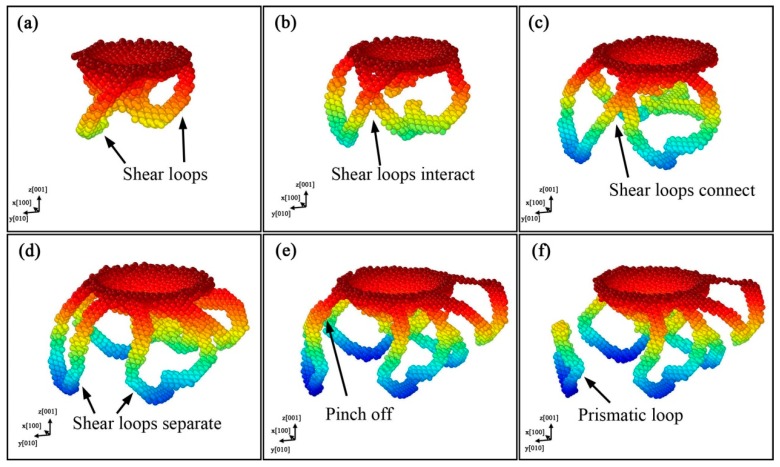
Formation of prismatic loops based on “lasso”-like mechanism for indentation on (001) plane, with colors representing depth from sample surface. The evolution process is illustrated on each subfigure from (**a**–**f**).

Figure 11 shows the formation process of prismatic loops, which is in accord with the “lasso”-like mechanism. It can be observed that the shear loops expand gradually with the indentation, because the edge components glide away from the indentation pit, and the screw components undergo limited cross-slip and attract each other, leading to the formation and emission of a new prismatic loop. We can also find that the shear loops form at the region of indentation pit, just like those in the indentation on AlN [25]. The difference lies in that two shear loops approach and interact with each other, and subsequently separate again, as shown in Figures 11b–d. This is because the screw components in contact have the same signs, resulting in repelling force and separation behavior. The following process is the same as the “lasso”-like mechanism. The screw components of one shear loop merge and pinch off, and then a prismatic loop forms, as shown in Figure 11e,f. 

**Figure 12:**
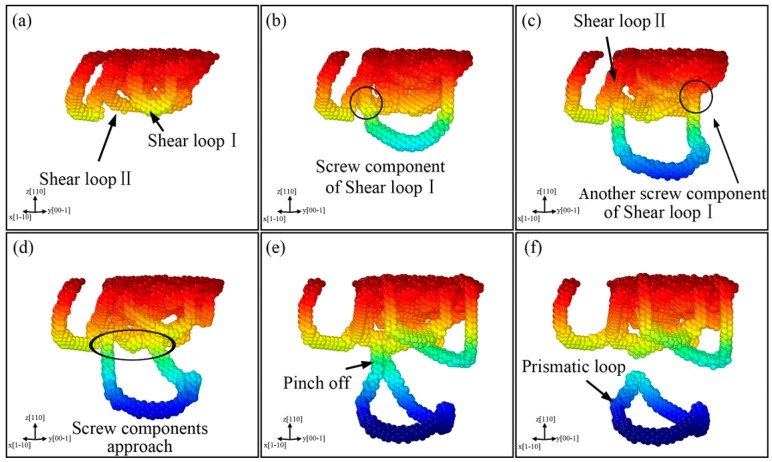
Formation of prismatic loops based on extended “lasso”-like mechanism for indentation on (110) plane, with colors representing depth from sample surface. The evolution process is illustrated on each subfigure from (**a**–**f**).

Figure 12 displays the results for indentation on the (110) surface, where the formation of prismatic loops is different from that on the (001) plane and can be called extended “lasso”-like mechanism. Figure 12a shows that Shear loop I and Shear loop II extend in the sample because of the motion of edge components. With the proceeding of indentation, the screw components of Shear loop I undergo cross-slip, then a screw component of Shear loop I glides on Shear loop II (Figure 12b) and the other one also approaches to Shear loop II (Figure 12c). 

**Figure 13:**
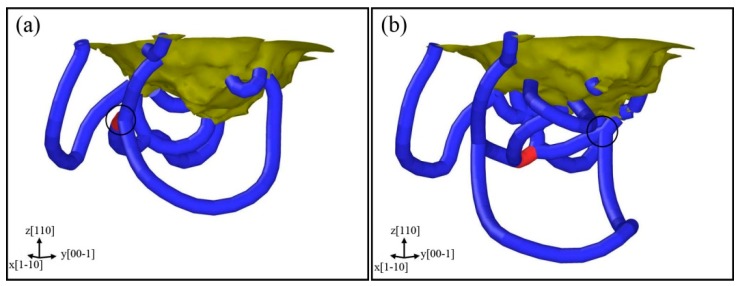
Dislocation structures analyzed with the dislocation extraction algorithm (DXA), where (**a**,**b**) are corresponding to Figure 12b,c, with black circles representing portions where two loops intersect with each other.

To display this process more clearly, the DXA analysis is also shown in Figure 13, where (a) and (b) correspond to Figure 12b,c, respectively. It can be observed clearly that the screw components of a shear loop glide on the other one, and the black circle zones indicate the positions where the two loops intersect. The two screw components of Shear loop I approach each other on Shear loop II (Figure 12d). Finally, the two screw components merge and pinch off, forming a new prismatic loop that is emitted from Shear loop II, as shown in Figure 12e,f. 

### 3.4. Dislocation Density

**Figure 14:**
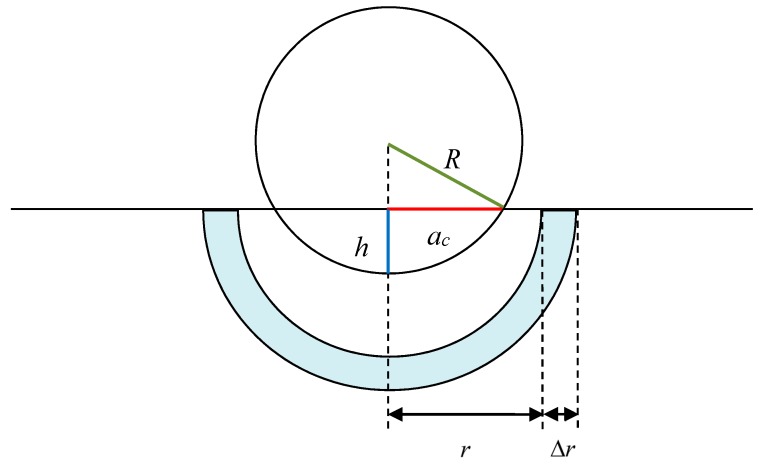
Schematic to evaluate the dislocation density.

Dislocation density is defined as the total length of dislocation lines in unit volume. Dislocation density is an important input in many hardening models, e.g., the famous Taylor model [43]. The schematic to evaluate the dislocation density in the sample under indentation is shown in Figure 14, where *a_c_* is the radius of the projected area, which can be calculated with

(13) $$a_{c}=\sqrt{R^{2}-{\left(R-h\right)}^{2}}$$

where *R* is the indenter radius and *h* the penetration depth. 

Assuming *R_p_* is the radius of the plastic zone, the corresponding volume can be calculated as described in [44],

(14) $$V_{p}=\frac{2}{3}\pi R_{p}^{3}-V_{ind}$$

where $V_{ind}=\pi h^{2}\left(R-h/3\right)$ is the imprint volume of indenter. Once the total length of dislocation lines, *L_d_*, is obtained, dislocation density in plastic zone can be derived with

(15) $$\rho =\frac{L_{d}}{V_{p}}$$

It must be noted that the local dislocation density, ρ(r), is varied with the distance from indenter. If the local dislocation density in hemispherical shells is determined, as shown in Figure 14, the dislocation density in plastic zone can also be obtained as in [45],

(16) $$\rho =\frac{1}{V_{p}}\int_{a_{c}}^{R_{p}}4\pi r^{2}\rho (r)dr$$

**Figure 15:**
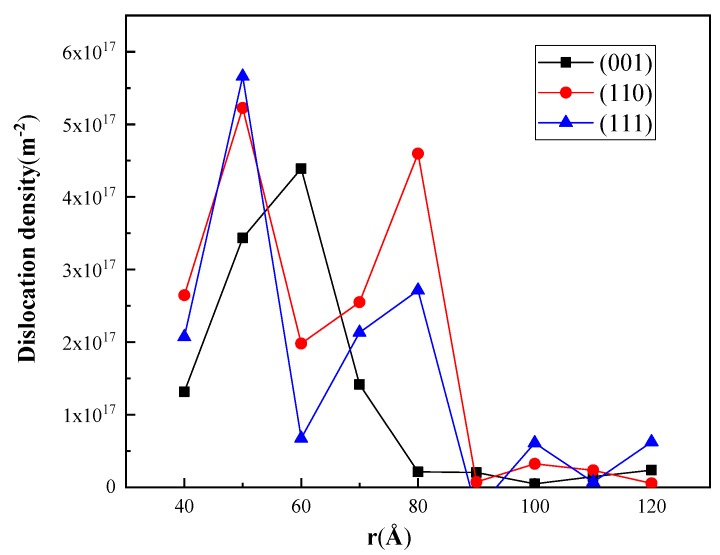
Local dislocation densities in samples indented on different planes after unloading.

In our simulation, *a_c_* ≈ 40 Å, and the length of dislocation lines can be obtained by DXA. Figure 15 shows the local dislocation densities in hemispherical shells with thickness Δr=0.5 Å at different positions, where the dislocation density varies with the indentation orientations. In general, the maximum values in the three curves appear at *r* = 50–60 Å, which is credible because Fischer-Cripps et al. also found that dislocation nucleation is related to the maximum shear stress, which appears at about 0.5*a_c_* beneath the indentation pit [46]. 

Considering the concepts of geometrically necessary dislocations (GNDs) and statistically stored dislocations (SSDs), the SSDs, such as dislocation dipoles and loops, will move in the material and do not induce strain gradients, whereas the GNDs need to accommodate the crystal curvature that arises with a non-uniform plastic deformation. Generally speaking, GND is related to strain gradients and SSD is related to equivalent plastic strain [47]. Alhafez et al. divided the dislocations in a crystal under indentation into two groups, the dislocations adhering on indentation pit and the closed dislocation loops that have been pushed out [48]. Obviously, the former is related to GND and latter to SSD. Thus, the dislocation density in the plastic zone is mainly the GND density.

The size factor of the plastic-zone, defined as $f=R_{p}/a_{c}$, is an important parameter and has been studied for various materials [49,50]. The radius of the plastic zone, *R_p_*, is set as the largest distance from a dislocation line bonding the pit to the center of the indenter, which can be determined by the IDS algorithm. In our work, we found that *R_p_* for indentations on (001), (110), and (111) planes were 97.7 Å, 111.7 Å, and 150.7 Å, respectively, and the corresponding f in B3-GaN are 2.4, 2.8, and 3.8, which was similar to those in fcc metals [51]. 

## 4. Conclusions

The mechanical property and microstructural deformation of B3-GaN films under indentation were studied using MD simulation, from which the following conclusions can be drawn.

The *P*-*h* curve of the indentation on the GaN (001) surface reveals that the pop-ins are related to dislocation nucleation, and the generation of many dislocations will induce the zigzag segment of the curve.

- The *P*-*h* curves for the indentation on the three planes exhibit anisotropy and show different depths at the onset of plasticity. (111) plane has the maximum hardness. The dislocation distributions analyzed by DXA and IDS show that only perfect dislocation with Burgers vector **b** = 1/2<110> can be found.
- Two mechanisms for the formation of prismatic loops were found. The “lasso”-like mechanism reveals that the screw components of a shear loop can attract each other and annihilate by cross-slip, finally a prismatic loop is formed. The extended “lasso”-like mechanism shows that two screw components of a shear loop can glide on another shear loop, and eventually a prismatic loop is also formed.
- The dislocation densities are different in the samples under the indentations on different oriented surface, and the corresponding size factors of the plastic zone in B3-GaN are similar to those in the fcc metals.

## Figures and Tables

**Table 1:** Properties of B3-GaN obtained from molecular dynamics (MD) and Exp./Cal. (Exp. and Cal. represent the results from the experiments and density functional theory (DFT), respectively).

Property	MD	Exp./Cal.	Error
Bulk modulus (GPa)	205.79	201.00 ^a^	2.3%
Lattice constant (Å)	4.51	4.5 ^b^	0.2%
Cohesive energy (eV)	4.34	4.32 ^c^	0.4%
C11(GPa)	318.21	296.00 ^d^	7.5%
C12(GPa)	149.58	152.00 ^e^	1.6%
C44(GPa)	122.83	155.00 ^f^	20.7%

^a^ [19], ^b^ [20], ^c^ [21], ^d^ [22], ^e^ [23], ^f^ [24].
